# Acute Pancreatitis Following Diagnostic Oesophagogastroduodenoscopy: A Rare Post-procedure Complication

**DOI:** 10.7759/cureus.95520

**Published:** 2025-10-27

**Authors:** Smriti Chaudhary, Pranav Jha

**Affiliations:** 1 Acute Medicine, Imperial College London, London, GBR; 2 Gastroenterology, Cambridge University Hospitals NHS Trust, Cambridge, GBR

**Keywords:** acute pancreatitis, ampullary biopsy, diagnostic endoscopy, iatrogenic complication, magnetic resonance cholangiopancreatography, oesophagogastroduodenoscopy (ogd), postprocedural pancreatitis

## Abstract

Acute pancreatitis is a recognised complication of endoscopic retrograde cholangiopancreatography (ERCP), but its occurrence after diagnostic oesophagogastroduodenoscopy (OGD) is exceedingly rare. We report a case of a woman who developed acute pancreatitis shortly after a diagnostic OGD during which a small peri-ampullary mucosal prominence was inadvertently biopsied. She presented with acute epigastric pain and was found to have imaging features consistent with pancreatitis in the absence of gallstones, alcohol use, or other causes. The patient was managed conservatively with intravenous fluids, analgesia, and gradual reintroduction of diet, leading to full clinical and radiological recovery. This case highlights an uncommon but important iatrogenic complication of diagnostic endoscopy and underscores the need for caution when performing biopsies near the ampulla of Vater, where minor trauma may precipitate transient pancreatic duct obstruction and pancreatitis.

## Introduction

Oesophagogastroduodenoscopy (OGD) is considered a safe and routine diagnostic procedure, with serious complications being exceedingly uncommon. In contrast, acute pancreatitis is a well-recognised adverse event following endoscopic retrograde cholangiopancreatography (ERCP), occurring in up to 10% of unselected patients and as high as 30% in high-risk groups [1]. However, pancreatitis after diagnostic OGD, without cannulation or contrast injection, is exceptionally rare and has been reported only sporadically in the literature [2].

In such cases, the proposed mechanism involves mechanical or thermal injury to the ampullary region, leading to transient oedema or obstruction of the pancreatic duct. This phenomenon has been described following ampullary biopsy, where even minimal manipulation near the ductal orifice may provoke pancreatic inflammation [3,4]. Although most reported cases resolve with conservative management, severe complications can occur.

Current endoscopic practice guidelines highlight the importance of avoiding trauma to the ampulla of Vater during diagnostic procedures and recommend that biopsies in the peri-ampullary region be performed cautiously or avoided unless clinically indicated [5].

We present a rare case of acute pancreatitis developing after a routine diagnostic OGD in which an unintentional peri-ampullary biopsy was taken, illustrating a preventable iatrogenic mechanism and emphasising the importance of vigilance in post-endoscopic abdominal pain.

## Case presentation

**Figure 1:**
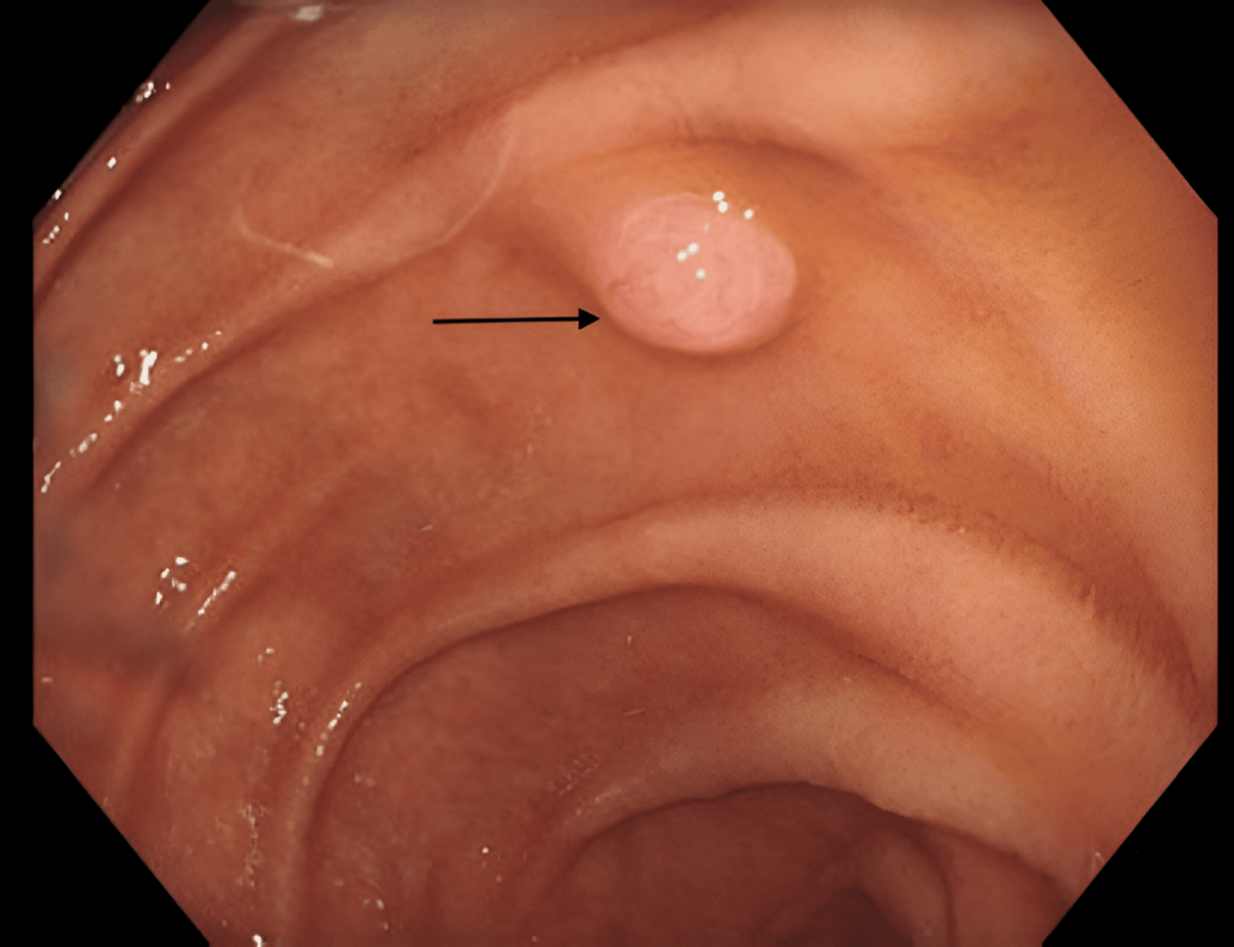
Endoscopic view of the peri-ampullary region showing a small mucosal prominence (arrow) that was mistaken for a polyp

**Figure 2:**
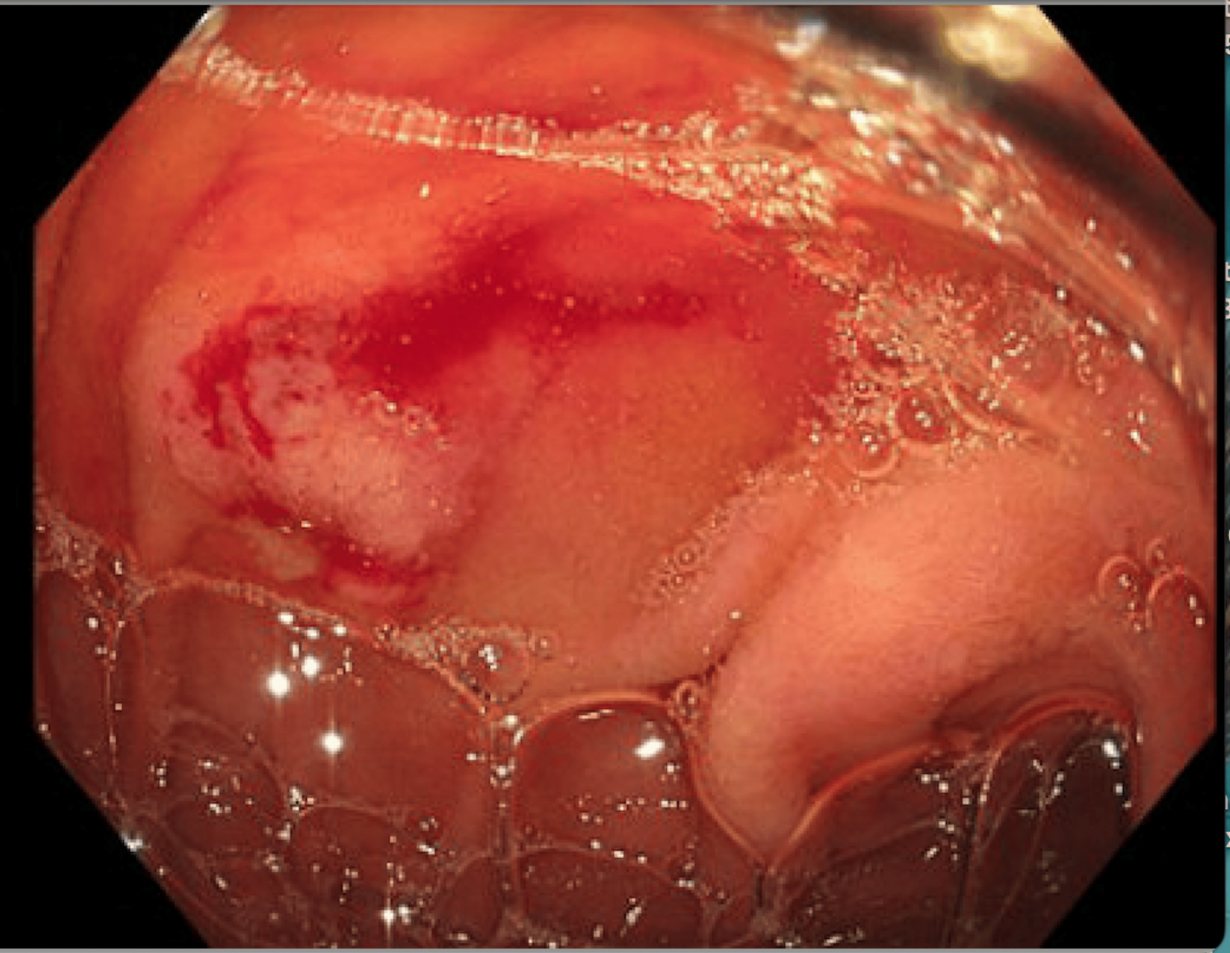
Post-biopsy appearance

A 77-year-old woman with a history of osteoarthritis and prior joint arthroplasties underwent a diagnostic OGD for evaluation of progressive dysphagia. During the procedure, several mucosal biopsies were obtained, including an unintentional peri-ampullary biopsy of a small mucosal prominence that appeared endoscopically similar to a duodenal polyp (Figure 1 ). This was subsequently biopsied (Figure 2).

Within several hours of the procedure, the patient developed severe epigastric pain radiating to the back, accompanied by nausea and vomiting. On presentation to the emergency department, her serum amylase level was markedly elevated at over 5,000 U/L. The blood results are summarised in Table 1.

**Table 1:** Trend of key laboratory parameters during hospital admission All results are expressed in standard SI units. mmol/L = millimoles per litre; µmol/L = micromoles per litre; U/L = units per litre; g/L = grams per litre; ×10⁹/L = 10⁹ cells per litre; mg/L = milligrams per litre; H- high (above reference range); L- Low (Below reference range)

Parameter	Reference Range	On Admission	Peak/Day 2–3	Recovery/Day 5–6	Pre-Discharge	Review in clinic (5 months after admission)	Interpretation
Serum sodium (mmol/L)	135–145	135	133	138	138	136	Stable throughout admission
Serum potassium (mmol/L)	3.5–5.0	3.2 (L)	3.1 (L)	3.7	4.5	4.5	Mild transient hypokalaemia corrected with fluids
Serum creatinine (µmol/L)	45–90	47	42	40	49	52	Normal renal function preserved
Adjusted calcium (mmol/L)	2.15–2.55	2.3	2.27	2.55	2.61	89	Mild transient fluctuation, normalised
Phosphate (mmol/L)	0.8–1.5	0.97	0.57 (L)	0.85	0.78	—	Transient hypophosphataemia resolved
Magnesium (mmol/L)	0.7–1.0	0.74	0.63 (L)	0.78	0.79	—	Slightly low during the acute phase
Alkaline phosphatase (U/L)	30–120	76	197 (H)	200 (H)	189 (H)	70	Reactive rise with normalisation showing a transient obstructive pattern that self-resolved
Albumin (g/L)	35–50	37	32	30 (L)	30 (L)	38	Hypoalbuminaemia of acute inflammation
Amylase (U/L)	25–125	5364 (H)	1681 (H)	—	—	—	Markedly elevated on admission, rapidly declined
Alanine transaminase (U/L)	0–40	51 (H)	53 (H)	58 (H)	59 (H)	15	Mild transient elevation only
Total bilirubin (µmol/L)	3–21	8	6	5	5	7	Normal, no biliary obstruction
C-reactive protein (mg/L)	<5	131 (H)	292 (H)	135 (H)	62 (H)	<4	Peaked early, declining with recovery and eventually normalised
White blood cell count (×10⁹/L)	4.0–10.0	10.6 (H)	13.8 (H)	11.3 (H)	6.1	5.2	Early leukocytosis resolved with improvement
Neutrophil count (×10⁹/L)	2.0–7.5	9.4 (H)	12.1 (H)	10.1 (H)	4.4	3.58	Neutrophilia resolving by discharge
Haemoglobin (g/L)	115–160	116	103 (L)	129	138	124	Transient mild anaemia improved
Platelet count (×10⁹/L)	150–400	372	412 (H)	396	491 (H)	297	Reactive transient thrombocytosis
LD (U/L)	125–250	—	288 (H)	—	—	—	Mild elevation during acute inflammation
Triglycerides (mmol/L)	<1.7	0.85	—	—	0.85	—	Normal; excludes hypertriglyceridaemia as a cause of pancreatitis

**Figure 3:**
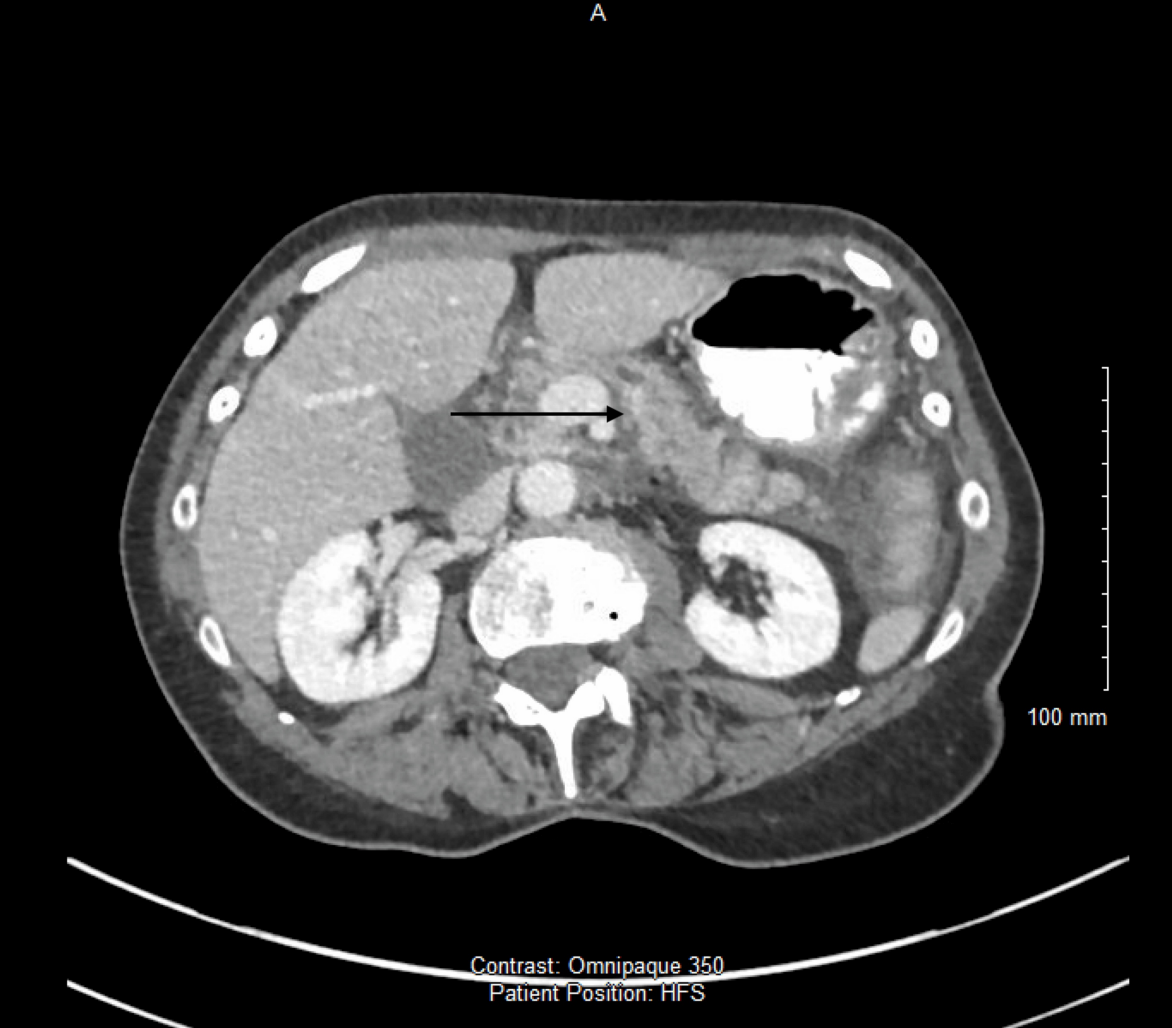
Axial contrast-enhanced CT image of the upper abdomen demonstrating peripancreatic fat stranding and inflammatory changes centred on the pancreatic head and second part of the duodenum (arrow)

**Figure 4:**
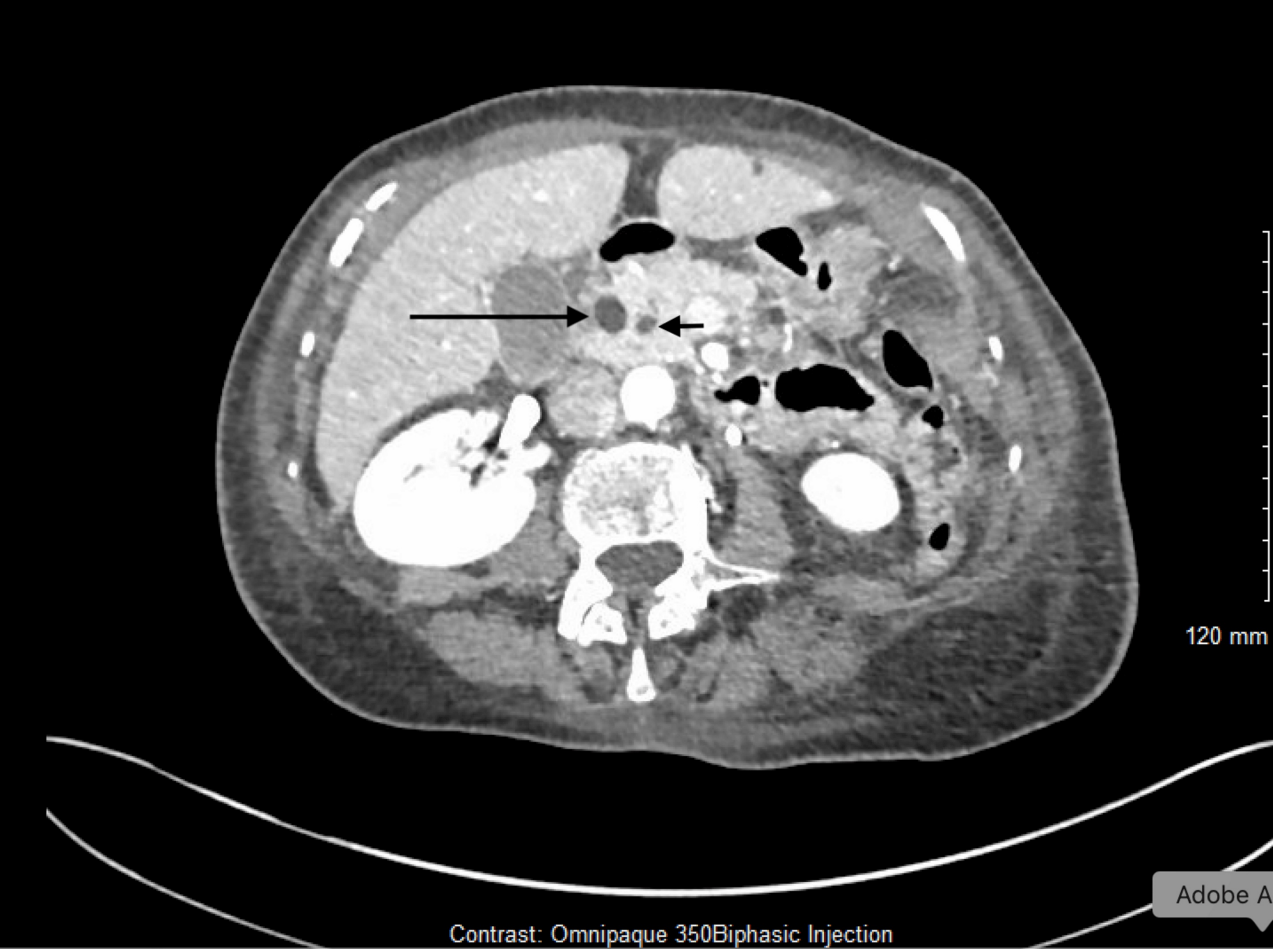
Double duct sign showing a dilated CBD (longer arrow) and pancreatic duct (shorter arrow) CBD: common bile duct

Contrast-enhanced computed tomography (CT) of the abdomen demonstrated inflammatory changes centred on the second portion of the duodenum with extension into the retroperitoneum, but no perforation, fluid collection, or free gas (Figure 3). A double duct sign was seen (Figure 4). These findings, combined with the typical clinical features and biochemical evidence, fulfilled the Revised Atlanta Criteria for acute pancreatitis [6].

**Figure 5:**
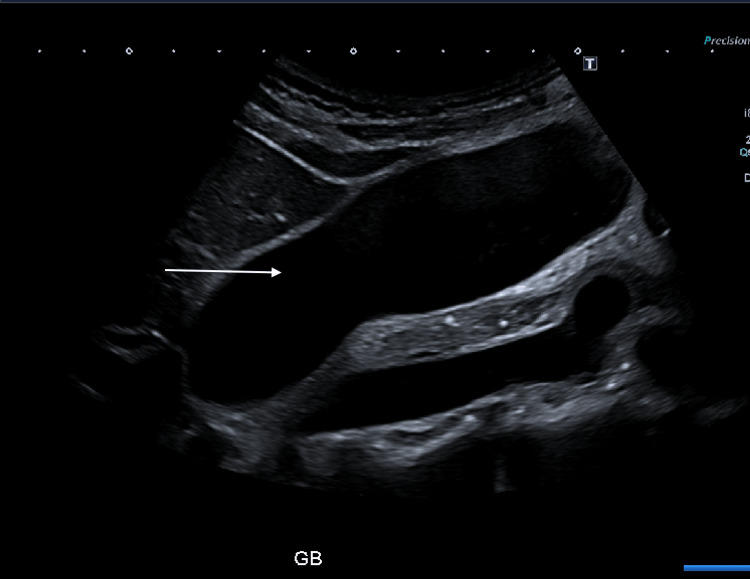
Longitudinal ultrasound image of the gallbladder showing a markedly distended, thin-walled gallbladder (arrow) without gallstones or pericholecystic fluid, consistent with reactive gallbladder distension secondary to acute pancreatitis

A right upper quadrant ultrasound performed early in admission demonstrated a distended gallbladder (Figure 5) with a small volume of sludge but no gallstones, and a common bile duct measuring 7 mm without intrahepatic ductal dilatation. These findings effectively excluded gallstone-related or obstructive aetiology. The patient was managed conservatively with intravenous fluids, opioid-based patient-controlled analgesia, and gradual reintroduction of oral intake with a low-fat diet. Her symptoms and inflammatory markers improved progressively.

**Figure 6:**
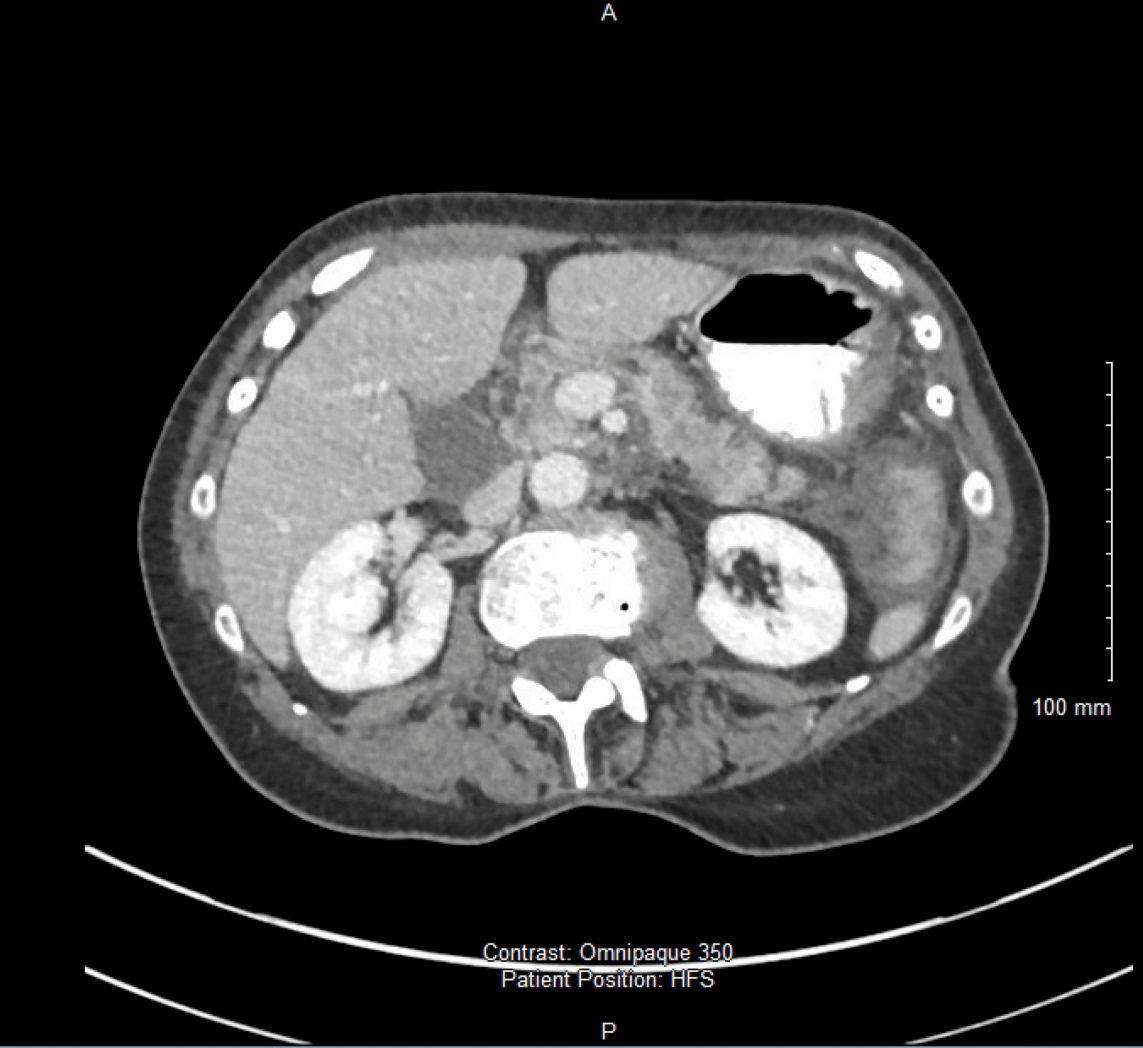
Axial contrast-enhanced CT image of the upper abdomen on follow-up showing resolution of peripancreatic inflammatory changes with normal pancreatic enhancement and clear peripancreatic fat planes, consistent with recovery from acute interstitial pancreatitis

A follow-up CT scan obtained during recovery demonstrated improvement in peripancreatic inflammation (Figure 6) but persistence of a mild “double-duct” sign, characterised by concurrent dilation of the bile and pancreatic ducts. In the context of clinical improvement and absence of a mass lesion, this finding was attributed to transient post-inflammatory change rather than malignancy, consistent with published evidence that the double-duct sign lacks specificity for neoplastic obstruction [7].

Histopathological analysis consisted of a cross-cut fragment of mucosa and submucosa containing scattered mildly dilated glands lined by low columnar epithelium within strands of smooth muscle and chronic inflammation in keeping with an ampullary biopsy [8]. There was no cytological atypia. No parasites, granulomata, dysplasia or evidence of malignancy were seen.

**Figure 7:**
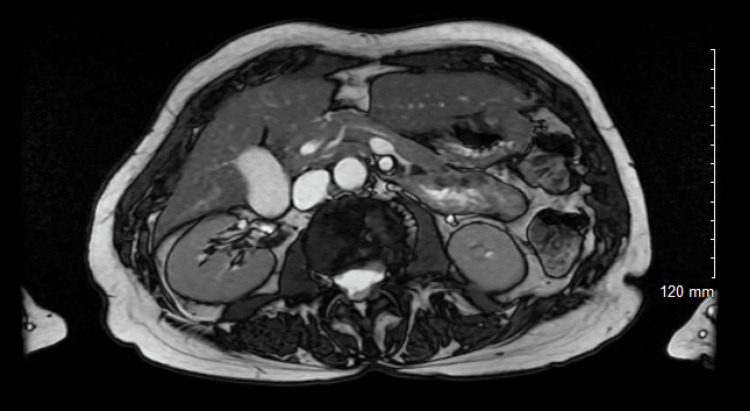
Axial MRCP image of the upper abdomen demonstrating a homogeneous pancreas with no residual peripancreatic inflammation or fluid collections MRCP: magnetic resonance cholangiopancreatography

When reviewed in the clinic four months after the initial presentation, her abdominal pain had subsided completely. A magnetic resonance cholangiopancreatography (MRCP) was performed to assess for a persistent ductal abnormality. The study demonstrated complete resolution of peripancreatic inflammation, no biliary dilatation, and no choledocholithiasis (Figure 7). The main pancreatic duct could be traced continuously to the ampulla without a stricture or transition point, confirming the absence of ongoing obstruction. MRCP was selected as a non-invasive imaging modality to exclude residual biliary or pancreatic ductal pathology, in line with best practice for follow-up of idiopathic or post-procedural pancreatitis [9].

The close temporal association with endoscopy, absence of other identifiable causes, and full radiological resolution supported a diagnosis of post-endoscopic (iatrogenic) acute pancreatitis secondary to unintentional peri-ampullary biopsy causing transient obstruction or oedema at the pancreatic duct orifice. The patient made a complete recovery with conservative management and remains under outpatient follow-up.

## Discussion

Acute pancreatitis following diagnostic OGD is an exceptionally rare event. Most iatrogenic pancreatitis cases are linked to endoscopic retrograde cholangiopancreatography (ERCP), where instrumentation or contrast injection can cause papillary oedema or direct ductal injury [10]. In contrast, pancreatitis after diagnostic endoscopy is uncommon and typically results from inadvertent trauma or manipulation near the ampulla of Vater. Only a few such cases have been documented, the earliest describing acute pancreatitis following limited ampullary biopsy despite the absence of ductal cannulation [3,4].

The mechanism in these instances is thought to involve transient obstruction of the pancreatic duct due to localised oedema, haemorrhage, or spasm of the sphincter of Oddi. Even minimal trauma to the ampullary mucosa can impede pancreatic outflow, leading to elevated ductal pressure and premature enzyme activation. The ampulla represents a critical anatomic junction where small disruptions can have disproportionate physiological consequences. In the present case, the biopsy was performed on a small mucosal prominence subsequently identified histologically as a peri-ampullary myoepithelial hamartoma (adenomyoma), a benign lesion composed of glandular and smooth-muscle elements. These lesions may distort the local architecture of the ampulla and increase susceptibility to post-procedural oedema or inflammation [8].

Diagnosis of acute pancreatitis is based on the Revised Atlanta Classification, which requires two of three features: characteristic abdominal pain, elevation of serum amylase or lipase three times above the upper limit of normal, and imaging findings consistent with pancreatitis [6]. In this case, all criteria were met--severe epigastric pain, a serum amylase exceeding 5,000 U/L, and CT evidence of peripancreatic fat stranding centred on the pancreatic head. The absence of gallstones, alcohol use or metabolic triggers supports a direct iatrogenic aetiology.

The follow-up CT scan showed improving peripancreatic inflammation but a persistent “double-duct” sign, characterised by mild dilation of the common bile and pancreatic ducts. Although traditionally associated with peri-ampullary malignancy, the double-duct sign is not specific and may also be seen in benign inflammatory or obstructive conditions. Menges and colleagues demonstrated that transient ductal dilatation can occur secondary to inflammation or fibrosis rather than malignancy [7]. In our patient, MRCP confirmed smooth ductal tapering without a mass or abrupt cutoff, consistent with a benign post-inflammatory change.

Radiologic follow-up was essential to confirm resolution and to exclude ongoing obstruction. Contrast-enhanced CT remains the imaging modality of choice for assessing disease severity, while MRCP provides excellent non-invasive visualisation of ductal anatomy and patency. Comparative studies suggest MRCP offers diagnostic accuracy comparable to endoscopic ultrasound (EUS) for evaluating the biliary and pancreatic ducts and should be preferred when non-invasive imaging suffices [9]. In this case, MRCP confirmed complete recovery and ductal continuity, obviating further intervention.

The clinical course mirrored other reports of post-biopsy pancreatitis, which are generally mild and self-limiting with conservative management. Supportive therapy, including intravenous fluids, analgesia and gradual reintroduction of diet, typically results in full recovery within days. Severe complications, such as necrosis or organ failure, are exceedingly uncommon but have been reported in isolated cases [11]. Our patient improved steadily with conservative care, achieving biochemical and radiological resolution before discharge.

From a procedural perspective, this case highlights the importance of a cautious biopsy technique in the peri-ampullary region. Ampullary biopsies should only be performed when there is a clear suspicion of neoplasia, as the potential diagnostic benefit rarely outweighs the risk of inducing pancreatitis. When required, biopsies should be shallow and taken tangentially, avoiding direct trauma to the papillary orifice. The European Society of Gastrointestinal Endoscopy (ESGE) advises that EUS may be preferable to direct biopsy for assessing submucosal or ampullary lesions [5]. Recognising benign entities, such as adenomyoma, can prevent unnecessary sampling and the associated risk of pancreatitis.

Overall, this case underscores that even diagnostic endoscopy can occasionally result in iatrogenic pancreatitis if the ampulla of Vater is inadvertently disturbed. Awareness of this possibility, judicious procedural technique, and appropriate follow-up imaging are key to prevention and early detection. When it occurs, the prognosis is excellent with conservative management, and complete recovery is expected once the inciting insult resolves.

## Conclusions

Acute pancreatitis following diagnostic oesophagogastroduodenoscopy is an exceptionally rare complication, most often resulting from inadvertent manipulation or biopsy of the ampulla of Vater. The mechanism likely involves transient pancreatic duct obstruction due to localised oedema or inflammation at the papillary orifice. This case emphasises the importance of endoscopists being aware that even seemingly benign ampullary lesions, such as adenomyoma, can predispose to post-procedural pancreatitis if biopsied. Careful case selection, a gentle technique and the use of endoscopic ultrasound for evaluation before biopsy can minimise risk. Prompt recognition and conservative management lead to complete recovery in most cases, underscoring both the rarity and excellent prognosis of this complication.
